# Supplementary material for: Modeling forest landscape futures: Full scale simulation of realistic socioeconomic scenarios in Estonia
Source: PLoS One. 2023 Nov 17;18(11):e0294650. doi: 10.1371/journal.pone.0294650 (PMC10655990; doi:10.1371/journal.pone.0294650)
Supplement: S1 Fig — (PDF) [file pone.0294650.s001.pdf]

S1 Figure. TWO EXAMPLES OF HARVEST PROBABILITY CALCULATION WITH HYPOTHETICAL CADASTRAL UNITS (COMPRISING 8 STANDS EACH) IN NEXTSTAND SIMULATION

Modeling forest landscape futures: full scale simulation of realistic socioeconomic scenarios in Estonia

Ants Kaasik, Raido Kont, Asko Lõhmus

Example 1. Intensive management

STAND 1: restriction class A maturity age + 10 p=0.2	STAND2: strictly protected maturity age + 5 p=0	STAND 3: unrestricted maturity age + 10 p=0.385	STAND 4: unrestricted maturity age + 20 p=0.385
STAND 5: unrestricted below maturity age p=0	STAND 6: unrestricted below maturity age p=0	STAND 7: unrestricted maturity age + 10 p=0.385	STAND 8: unrestricted below maturity age p=0

AGE ADJUSTMENT

p*=0.3	p*=0	p*=0.5775	p*=0.77
p*=0	p*=0	p*=0.5775	p*=0

RETENTION OF NON-ZERO PROBABILITIES

p*=0.3		p*=0.5775	p*=0.77
		p*=0.5775	

AVERAGING

Cadastral unit harvest probability
0.56625

EXPLANATION:
Each small box represents a stand in a cadastral unit

Information represented for each stand:
1) restriction zone
2) age relative to the maturity age (in years)
3) yearly clear-cutting base probability p
(based on the REAL scenario)

EXPLANATION:
Each stand yearly base probability p
is adjusted for relative stand age

Adjusted probability is denoted by p*

linear increase; exceeding the maturity
age by 20 years doubles the probability

EXPLANATION:
Only non-zero adjusted probabilities
are used for calculating the harvest
probability of the cadastral unit

EXPLANATION:
Averaging the probabilities from
previous step leads to
the cadastral unit harvest probability
for the ongoing simulation year

Example 2. Non-intensive management

STAND 1: restriction class B maturity age + 20 p=0.055	STAND 2: unrestricted maturity age + 10 p=0.055	STAND 3: unrestricted below maturity age p=0	STAND 4: unrestricted maturity age + 20 p=0.055
STAND 5: unrestricted below maturity age p=0	STAND6: strictly protected maturity age + 15 p=0	STAND7: strictly protected below maturity age p=0	STAND 8: unrestricted below maturity age p=0

AGE ADJUSTMENT

p*=0.11	p*=0.0825	p*=0	p*=0.11
p*=0	p*=0	p*=0	p*=0

RETENTION OF NON-ZERO PROBABILITIES

p*=0.11	p*=0.0825		p*=0.11

AVERAGING

Cadastral unit harvest probability
0.10083
