# Supplementary material for: Modeling forest landscape futures: Full scale simulation of realistic socioeconomic scenarios in Estonia
Source: PLoS One. 2023 Nov 17;18(11):e0294650. doi: 10.1371/journal.pone.0294650 (PMC10655990; doi:10.1371/journal.pone.0294650)
Supplement: S2 Fig — (PDF) [file pone.0294650.s002.pdf]

S2 Figure. FROM 2022 AGE STRUCTURE TO 2050 AGE PROBABILITIES IN A 5×5 KM LANDSCAPE

Modeling forest landscape futures: full scale simulation of realistic socioeconomic scenarios in Estonia

Ants Kaasik, Raido Kont, Asko Lõhmus

**(a) Age structure of stands as of 1.1.2022**

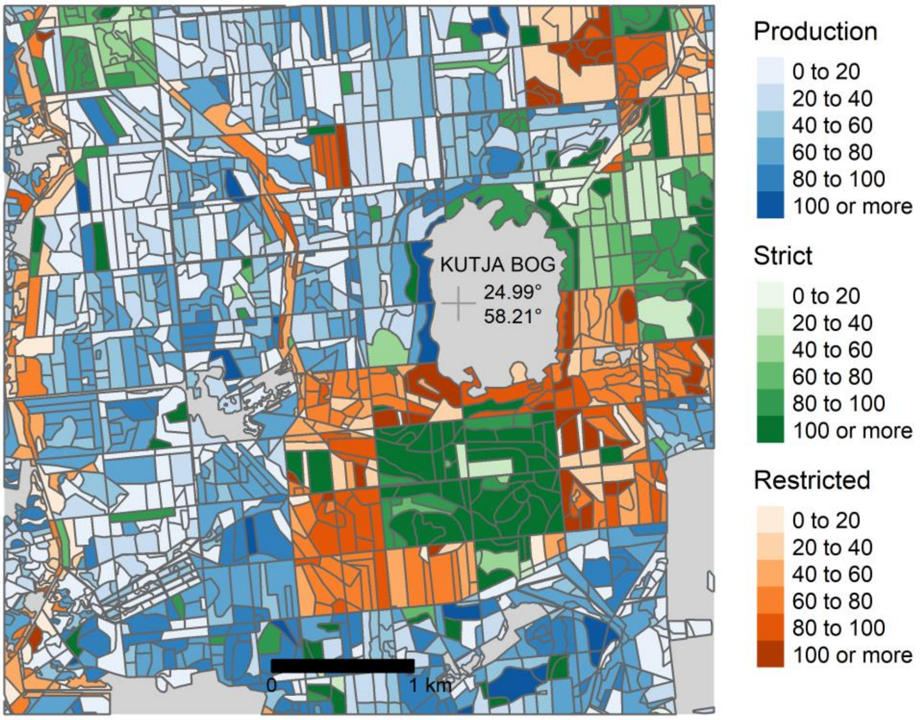


**(b) Probability of reaching >80 years of age in 2050**

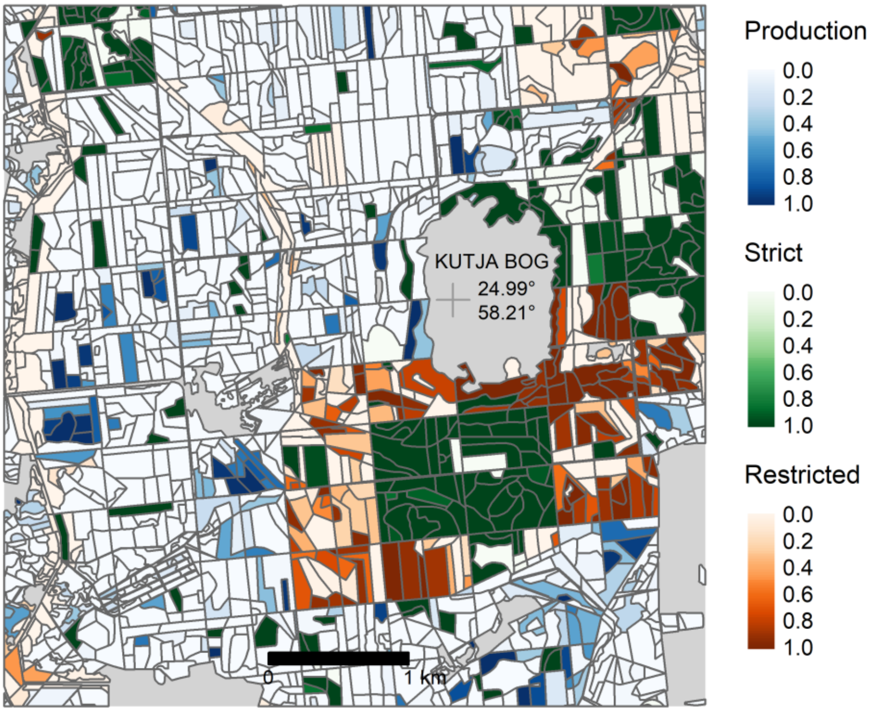

The example depicts a real landscape in southwestern Estonia, which includes all the main management types. The parcels on both graphs are stands (the basic units of simulation). The three main colors distinguish forests in the three main management zones; grey areas are non-forest. The color intensities depict: (a) mean stand age as of 1.1.2022; (b) the probability of being at least 80 years of age in 2050 according to the REAL scenario (50 simulation runs).

A currently typical Estonian protected-area zoning, with strict zones in the middle and restricted zones in the periphery, can be seen south from the Kutja bog. The orange stripes across the landscape are the restricted zones along stream banks. Most of the area is state-owned forest (with stands organized into regular quadrates); private forests of more irregular shapes can be seen, for example, in the southwest.

Informative changes from (a) to (b) include:

- the landscape will become more polarized (coarse-grained) in terms of the >80 year-old forest;
- the currently mid-aged **strict protected** area in the east will age and form a contiguous larger protected forest around the Kutja bog;
- **restricted-management zones** have the most variable performance, as seen from their geographical variation (e.g., the patch north vs southeast from Kutja bog), variable regimes (e.g., heavy harvesting pressure on the streamside forests) and intermediate probabilities being more frequent than for strictly protected or production forests;
- the main stepping stones remaining for old-forest biodiversity across the **production forest** are already currently strictly protected small patches (**woodland key habitats**); the likelihood of current production forest to survive 80 years of age is usually low and may occur as some larger patches (e.g., two patches in the southwestern part), usually because of higher allowed cutting age in poor-productivity sites.
