# Supplementary material for: Modeling forest landscape futures: Full scale simulation of realistic socioeconomic scenarios in Estonia
Source: PLoS One. 2023 Nov 17;18(11):e0294650. doi: 10.1371/journal.pone.0294650 (PMC10655990; doi:10.1371/journal.pone.0294650)
Supplement: S1 Table — (PDF) [file pone.0294650.s003.pdf]

S1 Table. DESCRIPTIONS OF THE MODEL PARAMETERS AND THEIR VALUES IN SCENARIOS.

Modeling forest landscape futures: full scale simulation of realistic socioeconomic scenarios in Estonia

Ants Kaasik, Raido Kont, Asko Lõhmus

			PARAMETER VALUE IN THE SCENARIO					
PARAMETER NAME	PARAMETER DESCRIPTION	UNIT	DEFa	DEFb	MODa	MODb	GOV	REAL
startyear	First year of the simulation	year	2022	2022	2022	2022	2022	2022
endyear	Final year of the simulation	year	2050	2050	2050	2050	2050	2050
alvarlimit	Maximum allowed clear-cut area in alvar sites	m2	20000	20000	20000	20000	24000	20000
waterbodylimit	Maximum allowed clear-cut area in riverine stands	m2	20000	20000	20000	20000	24000	20000
boglimit	Maximum allowed clear-cut area in bog sites	m2	50000	50000	50000	50000	60000	50000
defaultlimit	Maximum allowed clear-cut area in other stands	m2	70000	70000	70000	70000	84000	70000
juridical_prob	Probability of INT in juridical person owned estates		0.95	0.95	0.95	0.95	0.95	0.95
physical_prob	Probability of INT in physical person owned estates		0.35	0.35	0.35	0.35	0.35	0.35
intensive_prob	CUTPROB of INT private stands		0.35	0.385	0.35	0.385	0.385	0.385
nonintensive_prob	CUTPROB of non-INT private stands		0.05	0.055	0.05	0.055	0.055	0.055
nonintensive_prob_end	CUTPROB of non-INT private stands by year 2050		0.055	0.055	0.055	0.055	0.055	0.13
state_prob	CUTPROB of state-owned stands		1	1	1	1	1	1
nonintensive_to_intensive	Yearly proportion of private non-INT estates converted to INT		0	0	0.028	0.028	0	0.028
reforested_to_natural	Probability of a planted stand converting to a natural stand post-harvest		0.05	0.05	0.05	0.05	0.05	0.05
natural_to_reforested	Probability of a natural stand converting to a planted stand post-harvest		0.05	0.05	0.05	0.05	0.05	0.05
restrA_prob	CUTPROB of private stands in restriction class A		0.2	0.2	0.2	0.2	0.2	0.2
restrB_prob	CUTPROB of private stands in restriction class B		0.1	0.1	0.1	0.1	0.1	0.1
restr_state_prob	CUTPROB of state-owned stands in restriction classes A or B		0.05	0.05	0.05	0.05	0.05	0.05
state_fellinglimit	Yearly felling limit of state-owned stands	m2	120000000	120000000	120000000	120000000	120000000	120000000
second_to_upper	Proportion of the stock in the second layer added to upper layer yearly		0.01	0.01	0.01	0.01	0.01	0.01
clearage	Age below which the stand is considered a clear-cut	year	6	6	6	6	6	6
FHI_distance	Distance to a settlement below which state-owned stands are of HPI	m	1000	1000	1000	1000	1000	1000
FHI_age	Time past maturity age after which HPI stands are included for clear-cutting cluster determinations	year	20	20	20	20	20	20
ordinary_age	Time past maturity age after which state owned non-HPI stands are included for clear-cutting cluster determinations	year	10	10	10	10	10	10
unregistered_in	Year when all unregistered stands are included in management planning	year	2050	2050	2050	2050	2050	2050
