# Supplementary material for: Modeling forest landscape futures: Full scale simulation of realistic socioeconomic scenarios in Estonia
Source: PLoS One. 2023 Nov 17;18(11):e0294650. doi: 10.1371/journal.pone.0294650 (PMC10655990; doi:10.1371/journal.pone.0294650)
Supplement: S2 File — (DOCX) [file pone.0294650.s005.docx]

**S2 File. ALGORITHMS USED FOR UPDATING THE REGISTRY.**

**Modeling forest landscape futures: full scale simulation of realistic socioeconomic scenarios in Estonia**

**Ants Kaasik, Raido Kont, Asko Lõhmus**

**A: ASSIGNING SIMILAR NATURAL AND REFORESTED STANDS FOR REGISTERED STANDS**

Each registered forest subcompartment was assigned a similar (natural) registered forest subcompartment which had to have a reforestation rate of less than 20% and age of the stand more than 60 years. This was accomplished as follows:

1) If the subcompartment itself met the criteria then the stand itself was declared as its similar (natural) stand

2) If the subcompartment did not meet the criteria but its reforestation rate was less than 20% then a similar subcompartment was sought from 1km neighbors which had the same forest site type and same dominant tree species and met the criteria.

3) If no stands were available by 2) then the match of dominant tree species was dropped.

4) If no stands were available by 3) then the match of forest site type was also dropped.

5) If no stands were available by 4) then the steps 2) to 4) were repeated with the 1km neighborhood requirement dropped.

6) If the reforestation rate of the stand was 20% or more then a similar registered forest subcompartment was chosen from 1km neighbors which had the same forest site type and quality class and met the criteria.

7) If no stands were available by 6) then the match of quality class was dropped.

8) If no stands were available by 7) then the match of forest site type was also dropped.

9) If no stands were available by 8) then the steps 6) to 8) were repeated with the 1km neighborhood requirement dropped.

Each registered forest subcompartment was also assigned a similar (reforested) registered forest subcompartment which had to have a reforestation rate of 20% or more (this amounts to about 20% of the total number of registered stands). This was accomplished as follows:

1) If the subcompartment itself met the criteria then the stand itself was declared as its similar (reforested) stand

2) If the subcompartment did not meet the criteria then a similar subcompartment was sought from 1km neighbors which had the same forest site group type and same dominant tree species and met the criteria.

3) If no stands were available by 2) then the match of dominant tree species was dropped.

4) If no stands were available by 3) then the match of forest site group type was also dropped.

5) If no stands were available by 4) then the steps 2) to 4) were repeated with the 1km neighborhood requirement dropped.

**B: CREATING STAND BORDERS FOR THE FOREST LAND NOT PRESENT IN THE REGISTER**

The unregistered forest land was obtained by distinguishing the difference between the Estonian Basemap layer of woody vegetation (E_305_puittaimestik_a, type: “Forest”; provider: Estonian Land Board, 07.04.2021) and stand polygons in the national forest register (provider: Estonian Environmental Agency, 10.01.2022).

The following step by step intersections with different map layers (and the Voronoi tessellation in the latter part) were used to derive the stand borders and basic parameters:

1) Intersection with the cadastral units map layer (provider: Estonian Land Board, 21.10.2022).

2) The sequential creation of 10 m wide inside and outside buffer to remove the narrow (diameter < 20 m) polygons resulting in step 1 and 2.

3) Intersection with the map layer of parks (provider: Estonian Environmental Agency, 04.10.2022) and cemeteries (“E_301_muu_kolvik_ka”, type: “Cemetery”; provider: Estonian Land Board, 07.04.2021).

4) Intersection with town (type 3, 4 ,5 and 6) borders from the settlements map (provider: Estonian Land Board, 07.04.2021).

5) Removing all the polygons with size less or equal to 1.5 ha from further division and treating them as separate stands.

6) Intersection with coniferous forests (Corine Land Cover 2018, code: 312), the polygons that were not coniferous and had the size <=1.5 ha were removed from further division and treated as separate stands.

7) Intersection with mixed forests (Corine Land Cover 2018, code: 313), the polygons that were not mixed and had the size <=1.5 ha were skipped from further division and were treated as separate stands.

7) Intersection with soil map polygons containing peat (Kmoch et al. 2021, est_soiltype: M, S or R; the R and S polygons were pooled).

8) Removing small (size <= 0.1 ha) or narrow (diameter < 10 m, sequential creation of 5 m wide inside and outside buffer) polygons.

The Voronoi tessellation was used to divide the polygons from the preceding intersections to stands with similar shape and size as the registered stands. Random points were used as the centers for the tessellation and the polygons with more complex shape were divided to larger number of stands. To distinguish the initial polygons by shape the compactness index (comp. ind. = 4π*area/perimeter^2^) was calculated for each polygon. After visual checking, the index value 0.16 was chosen as the cutting point between the complex (comp. ind. <= 0.16) and compact polygons.

9) Polygons with size <= 5 ha and compactness index > 0.16 were skipped from further division and were treated as separate stands.

10) Polygons with size > 5 ha and compactness index > 0.16 were tessellated. The number of random points (n) was generated according to the formula: n = polygon area in hectares/5. The minimum allowed distance between the points was 100 m.

11) Polygons with size > 5 ha and compactness index <= 0.16 were tessellated. The number of random points was equal to the polygon area in hectares. The minimum allowed distance between the points was 100 m.

12) Polygons with size <= 5 ha and compactness index <= 0.16 were tessellated. The number of random points (n) was generated according to the formula: n = polygon area in hectares*2. The minimum allowed distance between the points was 25 m.

The following basic descriptive parameters were calculated for each stand:

13) The mean canopy height (m) from the lidar based canopy height model (years 2019-2021, resolution 4×4 m, provider: Estonian Land Board).

11) The mean crown cover (%) from the lidar based crown cover model (years 2018-2021, resolution 10×10 m, Arumäe and Lang 2018).

12) The soil type with the largest areal coverage from the soil map (Kmoch et al. 2021).

13) The forest type according to the overlap of the stand centroid with conifer, mixed or deciduous forest polygons of the Corine Land Cover 2018 map.

14) The stand protection status according to the overlap of the stand centroid with areas of either strict protection or restricted management regime (borders of the protected areas were obtained from the Estonian Nature Information System on 22.11.2022, maintainer: Estonian Environmental Agency).

The analysis was performed in R version 4.2.2 (R Core Team 2021) with the “tidyverse” (Wickham et al. 2019) package used for the overall data curation, “sf” (Pebezma 2018) for the spatial operations and “spatstat” (Adrian et al. 2015) for generating the random points. QGIS version 3.28.4 (QGIS Development Team 2023) was used for visually checking the data.

**C: CREATING FOREST COMPOSITION FOR THE FOREST LAND NOT PRESENT IN THE REGISTER**

After creating the borders for the unregistered forest subcompartments each of the newly-formed stands was then assigned a similar registered forest subcompartment. This was accomplished for each unregistered stand as follows.

1) Find all registered subcompartments with centerpoints in 1km range from the centerpoint of the focal unregistered subcompartment (1km neighbors).

2) Out of those, keep only the ones with same soil type and Corine type and with similar height and coverage (less than 20% absolute relative difference or less than 10 units absolute difference).

3) Calculate for each stand in the subset a) the sum of absolute relative differences of coverage and height from the focal unregistered stand and b) the sum of absolute differences of coverage and height divided by 50 and then set the similarity distance as the minimum of a) and b).

4) Select the registered subcompartment with smallest similarity distance as the similar stand for the focal unregistered subcompartment.

5) If the subset selected by steps 1) and 2) was empty, repeat the process from 1) to 4) by allowing soil type to be in the same group (instead of an exact match).

6) If after steps 1) to 5) there were still no suitable candidates then allow the range in step 1) to be 5km and repeat steps 1) to 5).

7) If after step 6) there were still no suitable candidates, allow larger differences in height and coverage (30% and 20 in relative and absolute differences, respectively) and repeat steps 1) to 6)

8) If after step 7) there were still no suitable candidates do not restrict anything with steps 2) and select the registered stand from the 5km range with most similar coverage as the similar registered forest subcompartment.

In our case step 6) was needed for about 10% of the unregistered stands and step 7) was needed for less than 1% of the unregistered stands.

Finally, the compositions for these newly formed stands were copied from their respective similar registered stands thus providing the starting register and the future natural register was also produced as before.

REFERENCES

Adrian Baddeley, Ege Rubak, Rolf Turner (2015). Spatial Point Patterns: Methodology and Applications with R. London: Chapman and Hall/CRC Press, 2015. <https://www.routledge.com/Spatial-Point-Patterns-Methodology-and-Applications-with-R/Baddeley-Rubak-Turner/9781482210200/>

Arumäe, T., & Lang, M. (2018). Estimation of canopy cover in dense mixed-species forests using airborne lidar data. European Journal of Remote Sensing, 51(1), 132-141.

Kmoch, A.; Kanal, A.; Astover, A.; Kull, A.; Virro, H.; Helm, A.; Pärtel, M.; Ostonen, I.; Uuemaa, E. (2021). EstSoil-EH: a high-resolution eco-hydrological modelling parameters dataset for Estonia. Earth Syst. Sci. Data, 13, 83-97. <https://doi.org/10.5194/essd-13-83-2021>

Pebesma, E. (2018). Simple features for R: Standardized support for spatial vector data. The R Journal, 10(1), 439–446. <https://doi.org/10.32614/RJ-2018-009>.

R Core Team (2021). R: A language and environment for statistical computing. Vienna, Austria: R Foundation for Statistical Computing. <https://www.R-project.org/>.

QGIS Development Team (2023). QGIS Geographic Information System. Open Source Geospatial Foundation. URL [http://qgis.org](http://qgis.org/)

Wickham H, Averick M, Bryan J, Chang W, McGowan LD, François R, Grolemund G, Hayes A, Henry L, Hester J, Kuhn M, Pedersen TL, Miller E, Bache SM, Müller K, Ooms J, Robinson D, Seidel DP, Spinu V, Takahashi K, Vaughan D, Wilke C, Woo K, Yutani H (2019). “Welcome to the tidyverse.” _Journal of Open Source Software_, *4*(43), 1686. doi:10.21105/joss.01686 <https://doi.org/10.21105/joss.01686>.
