# Supplementary material for: Modeling forest landscape futures: Full scale simulation of realistic socioeconomic scenarios in Estonia
Source: PLoS One. 2023 Nov 17;18(11):e0294650. doi: 10.1371/journal.pone.0294650 (PMC10655990; doi:10.1371/journal.pone.0294650)
Supplement: Attachment — Submitted filename: repsponse_to_reviewers.docx [file pone.0294650.s008.docx]

**PONE-D-23-20659**

**Kaasik et al.**

Modeling forest landscape futures: full scale simulation of realistic socioeconomic scenarios in Estonia

**Replies to reviewers**

We are very grateful for your positive attitude and constructive comments! Please see our responses (R) to each query (Q) below.

**Academic Editor**

**Q1.** I've received comments from two reviewers. I agree with their assessments that your manuscript is well written overall but would benefit from some revision, restructuring and additional clarifying text. I think Reviewer 1 is correct in saying that the Methods section seems a little thin in particular, so I recommend focusing on fleshing out methodological (i.e., modeling) aspects along the lines noted by both reviewers. I think there is definitely an audience that will be interested in your manuscript, so it's really just a matter of presenting your model and the chosen scenarios as clearly as possible. With these changes, I think that your manuscript should be suitable for publication.

**R1.** Thank you for this evaluation! We carefully dissected the reviewers’ comments and: (i) improved the Methods section by highlighting further key points from the model script, (ii) clarified the stand variables included in the input data and modified by the model, (iii) expanded the current scenario descriptions and explained the complexity of the climate change effects on the scenarios in Discussion, (iv) split Fig. S1 into a simplified graphical version and a textual example in the Methods; (v) added Fig. S2 to illustrate the predictions at the stand scale, and (vi) added new explanatory comments on the model script in the File S1. Please see R7, R9, R10 and R13 for details.

**Q2. /**l**inguistic corrections accepted**/ Line 186 - delete the period after "6.8%" *** Line 230 - "judgment" instead of "judgement" *** Line 464 - delete "the" before "Estonian" *** Line 494 - delete the unnecessary hyphen between "forest" and "structure" *** Line 498 - insert "a" before "land-sharing"

**R2.** Corrected as suggested.

**Q3.** Lines 188-190 - This sentence is a little unclear. I tried to figure out exactly what you were saying about the timing of the scans vs. clear-cuts but, honestly, I'm not sure I follow
**R3.** We rephrased to clarify that, since our goal was to have the (model start) situation as of 1.1.2022, also the most recent (2021) lidar flight (either in spring or summer) missed those clear-cuttings that were completed after the flight, in the second half of the year. The whole issue is that lidar data detects fresh clear-cuts but there are additional clearcuts after the lidar flights that can be simulated after we can estimate their approximate total area based on the addition rate that is calculated from differences between subsequent flights.

**Q4.** Line 266 - many readers may be unfamiliar with alvars given their limited geographical distribution.
**R4.** We specified both in the text (“Simulation procedures and key parameters”; 1^st^ paragraph) and in Table 2 that these are forests on thin calcareous soils that poorly regenerate after clear-cutting.

**Reviewer #1**

**Q5.** In this manuscript, the authors propose a spatially (stand-based) and temporally explicit forest landscape model (FLM) that predicts the future arrangement and status of forests in Estonia. The authors state in the first paragraph of the abstract that they will demonstrate the FLM by predicting forest composition in 2050, but the manuscript (and the second paragraph of the abstract) goes on to show that the FLM is more of a harvest choice model, as its predictions are only of forest age with a thematic resolution of clear-cut or undisturbed forest within a handful of management-type zones. This is still highly valuable, but it is far more specific than forest composition as a broad term.

**R5.** This seems to be a misunderstanding between what the model can do and what we present in this first demonstration of the model in the paper. Please note that the model, as explained in Sections “Model structure” and “Simulating stand dynamics” uses and generates data at the level of individual tree-layer elements at the stand scale. This allows analyzing spatial and temporal dynamics at the resolution of those elements. For the current paper, which primarily introduces the model principle and script, we chose the most likely ‘macro’ scenarios of harvests as of potentially general interest to a wider public (see also the Editorial assessment, Q1). In fact, spatial variables (such as isolation) and temporal dynamics are even broader terms than composition; thus we rephrased the start of Abstract, which now mentions [that we can analyze] “regional forest characteristics”, “at forest stand resolution”, “for realistic timber harvesting decisions” and “[described as] stand compositions”. See also R8.

**Q6** /**linguistic corrections/** In general, while the manuscript reads well (minor typographical errors, such as line 54 where “missing of landscape” can be “missing landscape”) … In general, I advise against using “will” statements for model predictions. The FLM is a predictive model, the limitations of which are outlined in the discussion section. “Are expected to” and “are predicted to” are more accurate. Similarly, the authors’ use of the term “forecast” is somewhat in contradiction to the scenario-based approach, unless the authors also attribute some sense of relative likelihood to each scenario. (The authors do report most on the REAL scenario, but I thought this was because the variation between scenarios was relatively low.)

R6. Thank you for these notes: we have now checked the text and replaced the ‘will/forecast’ statements with ‘predict/expected’ to expressions (it was probably most visible in the last paragraph of Discussion).

**Q7.** ... there is insufficient detail in the manuscript as it stands. I encourage the authors to expand upon the methods section in particular, both in walking a reader through their model process with more specific examples, and to better detail the scenarios (and why the results are focused only on one scenario). If possible, including a climate-model aspect into the scenarios would be immensely valuable, if only in the form of more details in the discussion. *** Scenario descriptions could use more detail, especially since most of the results focused only on one scenario. Making the -a and -b scenarios separate, only to show that there was little difference between them, strongly implies that they could be removed from the manuscript with no loss (the authors might simply note that in preliminary testing, the simulated updates had little impact on the results). In their place, it would be good to see a scenario or two showing climate impacts. For example, would increasing forest stress prompt landowners to harvest timber while they could, or would it prompt them to preserve what they can? Similarly, what laws might the Estonian government be likely to pass that might regulate harvest in such situations?
**R7.** Thank you for this feedback. In direct response, we rewrote the scenario descriptions and brought in an example of how the iterative process of assigning harvest probabilities works, which was previously depicted on Fig. S1 (and we reworked that Figure, as requested by Reviewer 2). We added our thoughts of the climate-change effects to Discussion, since those are probably very complex and, in our 28-year time frame, rather uncertain (‘on-off’ type, i.e., most likely there will be little effect, but can be a complex one).

Regarding decision-making and policies - there is currently no evidence of any harvest regulation or decision-making based on climate change in Estonia, so we consider it premature to introduce one into the model. Rather, we see this as a another scenario exploring both liberal policies with exotic fast-growing tree species and conservation policies based on native tree species and reduction of clear-cutting with central-planning methods (and possibly anything in between). We are currently analyzing also the recent harvesting behavior of real land-owners, which is providing us a better quantitative basis for the parameterization of the ‘harvest willingness’. Having had these future developments in mind, we have tried to keep this particular analysis as simple as possible.

**Q8.** While the authors do share the R script for their model, the manuscript does not sufficiently describe it. The only forest attributes explicitly considered here are age (used as a proxy for other ecosystem services) and connectivity, based on same-age corridors. Results are largely presented in terms of age classes, comparing the two years of 2022 and 2050 (it would be nice to have seen some trajectories, given the implication that this FLM runs at a yearly timestep). The first paragraph of the abstract, in which the FLM is said to predict the much broader class of forest composition, needs to be modified accordingly.

**R8.** Please see R1 for a list of our efforts to better describe the model. The age-related outputs were indeed of interest in the current study (Introduction, last paragraph), but the model is not limited to those (see also R5). Note also that Fig. 4 presents four points in time and Fig. 5 presents three; and it is clearly said that, computationally, the output of each simulation is a full ‘forestry registry’ with 2M stands for each year × no. of runs. To handle this amount of data requires specific queries and we here have made those queries based on age structure across the whole country and scenarios (Fig. 2) or spatial characteristics (Fig. 4), across forest owners and management regimes (Fig. 3), or distribution patterns (Fig. 5).

**Q9** The methods section in general feels thin. Figure 1 provides only a heuristic description of the FLM; an example tracking, say, a single stand through model iterations would be much more concretely informative. Given that the model only tracks clear cuts and stand age (is there an assumption that stand boundaries remain consistent throughout time?), I would like to see more detail on the calculation of forest age (since not all forests are even-aged stands) and how well the assumption of simply updating age with time holds.

**R9.** We added the following specification to the 1^st^ paragraph of Section “Simulating stand dynamics”. The model and the input data allow to specify unequal mortalities by stand components as annual changes in their abundance. However, for the transparency of the current demonstration of the model performance, we did not introduce those changes in the 28-year time perspective presented. Similarly, it is true that stand boundaries remain consistent and it is one of the main features that allows model optimization. However, technically it is not difficult to change those borders and, moreover, it would be reasonable to do this also iteratively, since it is likely that the borders will be changed in particular places, not randomly. Especially stable are even-aged systems where previous clear-cuttings determine even-aged stands that are the likely future clear-cuts, and the dominant changes in stand borders are either merging or splitting, so that the borders partly remain the same. These changes are still more convenient to model with a patch-based than grid-based spatial structure.

**Q10.** Although the authors note that the FLM is spatially explicit, there is not a single stand-level map or figure in the manuscript. This seems like a missed opportunity. I would also like to see a map of the various zones (intensively-managed, etc.); this would be helpful as well when interpreting the bar charts by panel.

**R10.** Thank you; this is a valid point, particularly for readers from different landscapes and management zoning systems. We have prepared a new Fig. S2 where one can see the future probabilities of >80 year-old stand in an actual Estonian landscape by management zone at the stand resolution. This figure now complements the regional scale shown on Fig. 5 (left panel) and we have linked them through a relevant comment in Results.

**Reviewer #2**

**Q11.** I recommend revising the organization of the arguments in the text, removing those that do not have a strong importance in the flow of the discussion to improve clarity. Some technical aspects of the simulators are not very clear, like if growth models are implemented, which, if any optimization procedure, is applied. Furthermore, the distinction among the scenarios is difficult to understand and consequently it is not so easy to compare the output of the simulator among scenarios.

**R11.** We rewrote the scenario descriptions, including their general rationale. Please see R1 for our other efforts to better describe the model. Specifically, note our clarification (R9) that the current model version included compositional change of different tree layers, but not volume growth models. The latter can be easily added, since the input data include the stock measurements (m^3^/ha) for the same tree fractions at the time of the field survey; however, a valid model should include both subsequent growth and removals (including natural mortalities). While the growth curves are relatively well developed for the Estonian even-aged stands, the removals require additional decision and disturbance parameters that were beyond our current scope.

**Q12.** I understand that the simulator is something in-between a landscape simulator and a classical process-based simulator, but I encourage the authors to define better if this is a Decision Support System/Tool or another type of hybrid system, to allow other authors to refer to it in the literature of this sector.

**R12**. A very relevant comment. Indeed, it can be used as a Decision Support Tool, notably for strategic and landscape planning over mid-term horizons. We added the relevant remarks to Abstract and Discussion. In fact, we have already discussed this with our Environmental Agency and State Forest Management Centre people and have their support in developing this, although no commitment yet.

**Q13.** I must admit that the specifications exposed in Figure S1 are obscure and not easy to understand, for example when referring to the concept of probability.

**R13.** We acknowledge that the original Figure S1 might be too complicated for a reader. We reformatted the Figure S1 into a more basic form and described its logic through an example in the main text.

**Q14.** Line 15 - Does this model has a name? It would be worth mentioning it in the abstract.

**R14.** It did not yet have a name but you are right - it is indeed a good idea for future reference to provide one. So, we named it ‘NextStand’, which refers both to the iterative process of stand consideration, the adjacency constraints, and the future state predicted in the simulation.

**Q15.** /**linguistic corrections/** Line 28 replace with „decreases its potential to fulfill biodiversity targets“ or „is a problem for biodiversity conservation“ *** Line 76 – replace „forecast“ with „simulate“ *** Line 76 – replace „process simulation“ with „dynamic process-based“ *** Line 77 – replace „uses“ with „is initialized with“ *** Line 78 – replace „is“ with „its simulations are“

**R15.** Corrected as suggested.

**Q16.** Line 31 - It is unclear what these units are and Line 32 - Is this spatial variability?

**R16.** We added “predictable” to (i); here we mean, for example, forest stands that can be subjected to iterative change in the simulation. The modeling becomes much more complex when the spatial units change all the time (see also R9). We improved (ii) as: “capture their variability of temporal change with simple ecological and socioeconomic (including human decision-making) variables.” In our case, such computationally simple, but conceptually complex, variable was the willingness to harvest by forest owners as addressed in Discussion.

**Q17.** Line 50 - What is meant here for state defense? This is not clear.

**R17.** Forests represent an important terrain obstacle for military vehicles and are important for providing concealment in military conflicts. Research on this is significant in forested countries (also in Estonia, e.g., Vennik & Lang, 2023 Forest and military aspect of terrain: forest height, canopy cover and visibility in Estonia. Estonian Journal of Military Studies). The Estonian Forest Act devotes a whole paragraph (§36) on training in forests.

**Q18.** Line 54 – replace with „the extent and complexity of“ with „large“?

**R18.** Corrected: “large and complex”.

**Q19.** Lines 101-104 - Are all these Roman numbers in parentheses really needed? *** Lines 102-103 - This sentence is unclear. Are you just interested in the variability / uncertainty among the predictions for scenarios for development and conservation? *** Line 104 - Which estimates?

**R19.** We agree that the questions part was not very clearly formulated and we rewrote it. That is, we are interested in the predicted forest compositions, and their variation in relation to key variables, which in turn were addressed by varying likely future scenarios of different complexity.

**Q20.** Lines 104-107 - This should go to the methods.

**R20.** Corrected (merged with 2^nd^ and 3^rd^ paragraph of “The study system and input data”).

**Q21.** Line 115 - Define a retention tree

**R21.** Retention tree is defined as any tree left permanently standing at regeneration felling.

**Q22.** Line 400 – does „factors“ mean „digits“ in this context?

**R22.** We meant “two reasons”: a lack of sampling error + improvement in precision of measurement errors present in each stand. We shortened this explanation for clarity.

**Q23.** Figure 1 „Assigning harvest events“- Is this where optimization takes place?

**R23.** Yes, it includes all the procedures of how the clear-cutting events were simulated, i.e., the spatial alternatives in their priority order, and the positive answers to the double question of harvest willingness and whether it is allowed. As a slight improvement to better comply with the figure, we used the word “assigned” in the first sentence of “Simulation procedures and key parameters”.

Thank you for your assistance,

Ants Kaasik

Raido Kont

Asko Lõhmus

25.08.2023
