# Supplementary material for: Modeling forest landscape futures: Full scale simulation of realistic socioeconomic scenarios in Estonia
Source: PLoS One. 2023 Nov 17;18(11):e0294650. doi: 10.1371/journal.pone.0294650 (PMC10655990; doi:10.1371/journal.pone.0294650)
Supplement: Attachment — Submitted filename: response_to_reviewers.docx [file pone.0294650.s009.docx]

**PONE-D-23-20659R1**

**Kaasik et al.**

Modeling forest landscape futures: full scale simulation of realistic socioeconomic scenarios in Estonia

**Replies to reviewers**

We are very grateful for your positive attitude and constructive comments! Please see our responses (R) to each query (Q) below.

**Academic Editor**

**Q1.** I have gone through your revised manuscript, as have the two original reviewers. I think it is nearly suitable for publication with some relatively modest changes. In particular, look at the comments from Reviewer 2, who noticed (as did I) that some of your reviewer responses didn't quite make it into the main text. (For example, it did seem odd to me that you mentioned optimization at least a few times in the abstract and Introduction, but it didn't really show up in the Methods section.) Such issues are relatively simple to fix, just check through the comments from Reviewer 2 and make sure they're represented in some way in the manuscript. I have a few comments of my own listed below (under Additional Editor Comments), but they're all minor.

The one other change that I'll ask you to make is to remove me, Adriano Mazziotta and the anonymous reviewer from your Acknowledgements section. Certainly, I appreciate it, but to ensure equitable recognition and avoid any appearance of partiality, the policy across PLOS journals is to not include editors or peer reviewers—named or unnamed—in the Acknowledgments.

Once you make these changes to your manuscript and submit the revised version, I should be able to make an editorial decision quickly.

**R1.** Thank you for this evaluation! We removed the sentence from the acknowledgements and improved the manuscript according to other specific comments (see below).

**Q2. /**l**inguistic corrections accepted**/ Lines 114-115 - "retain retention trees" seems weirdly redundant. Maybe "keep retention trees"? *** Line 163 - "were" instead of "was" ***

Line 166 - "include" instead of "includes" *** Line 183 - "were" instead of "was" *** Line 265 - Maybe "weighted" instead of "weighed"? *** Line 423 - Maybe "weighted" instead of "weighed"?

**R2.** Corrected as suggested.

**Q3.** Lines 186-187 - Assuming that the 1/4 of Estonia updated each year doesn't overlap with previous years, the 3 years of lidar data (2019-2021) gives you about 75% coverage of the country. Is that correct? Lines 187-195 - I'm still a little fuzzy on this. I think that I understand why the percentage of clear-cuts not present in the registry is higher in 2021 than in 2020 or 2019, given there is often a delay before owners enter a clear-cut in the registry. I also understand why the 8.4% of unregistered clear-cuts in 2021 is an underestimate since the scans don't capture cuts later in the year. I think the problem is the sentence that starts with "Thus, further 0.8%...". I don't see how this clarifies your estimation of the number (ca. 24000) of unrecorded clear-cuttings. Instead, isn't your point that the most recent lidar data will yield the highest percentage of detected, unregistered clear-cuts because of the delay in owner documentation/reporting?

**R3.** We now added a full description of the procedure in Supplementary file 3, with a reference to it and minor changes in the relevant text paragraph. Thus, in fact, each year about one half of Estonia is updated but this is done in two separate occasions (one quarter at a time), so that at the time of NextStand model development we had data for one quarter from 2021, for two quarters from 2020 and for one quarter from 2019.

The principal arithmetic for obtaining the estimate of approximately 24 000 stands, which were clear-cut by 1.1.2022 but not detected by the lidar scans available, is as follows. Based on the detection rates of 8.4% for 2021, 7.6% for 2020, and 6.8% for 2019, we assume that for those two quarters of Estonia that were scanned in 2020 the detection rate would also be 8.4% if the scans would have been carried out in 2021. Thus for those two quarters 0.8% of the clear-cut stands are still not detected. We also assume that for the quarter of Estonia that was scanned in 2019 the detection rate would also be 8.4% if the scan would have been carried out in 2021 and thus for this quarter 1.6% of the clear-cut stands are still not detected. This means that S=0.5*0.8%+0.25*1.6%=0.8% of all Estonian forest stands are not detected.

But given that the 8.4% rate for 2021 is actually based on roughly mid-year, this means that the actual rate for the end of 2021 would be 0.4% greater. This correction is missing from all quarters so we should add 0.4% to S and we get 1.2% as the final estimate. Multiplying this estimate with the number of stands present in the forest registry produces the estimate for the undetected stands.

**Reviewer #2**

**Q4**. ... in some cases the authors replied to my comments without taking specific actions on the manuscript. In Q17, a definition of state defense is provided to me but not for the general reader, who is supposed to know what it means.

**R4.** We have now added a sentence to the introduction (Line 51)

**Q5.** In Q22, the two factors are specified only in the reply but not described in the text.

**R5.** This is a misunderstanding as we have specified both factors in the text (Lines 438—440: “Compared to sample-based approaches our model precision is increased both because we sample the whole population and, because, due to central limit theorem, also the individual stand measurement errors “cancel out more effectively”.)”

**Q6.** In Q23, “where optimization is applied in the simulator in Fig. 1?”. The reply to the question does not imply any change in the Figure or in the methods. As optimization is involved in several aspects of the simulator, I would expect that all of them are listed either in the methods or in the figure as they are mentioned in R23.

**R6.** First, please note that the optimizations mentioned in the text are model performance optimizations, which include multiple aspects as explained in Methods and in the Supplements. We now summarize these aspects in Discussion, end of first paragraph: “The main features that allowed us to optimize the computation effort were modelling the whole process at a stand level (cf. regular grid-level), considering a stable adjacency structure of stands throughout and modelling the order of clear-cuts within a cadastral unit using the “largest stand first” approach.”

However, the last aspect is actually also connected to the optimization perspective of the forest owner. This modelling choice has empirical support in our data (clearcutting of the largest stand in a cadastral unit was ca ¼ more likely than clearcutting any other stand). We now mention this evidence in that sentence (Lines 286—289). (Of course, such an approach might not lead to maximal allowed cutting areas for the ongoing year when the total area is limited - e.g. with 7 ha total limit as in Estonia: when neighboring mature stands with areas 3, 4 and 5 ha, respectively, do not have any clearcut stands, using the largest-first optimization leads to clear-cutting of the 5 ha stand whereas clearcutting the two smaller stands instead would lead to a larger total).

Thank you for your assistance,

Ants Kaasik

Raido Kont

Asko Lõhmus

02.11.2023
